# (1*S*,3*R*,8*R*)-9-(1-Amino­ethyl­idene)-2,2-dichloro-3,7,7-trimethyl­tricyclo­[6.4.0.0^1,3^]undecan-10-one

**DOI:** 10.1107/S1600536811005307

**Published:** 2011-02-16

**Authors:** Ahmed Benharref, Essêdiya Lassaba, Daniel Avignant, Abdelghani Oudahmane, Moha Berraho

**Affiliations:** a Laboratoire de Chimie Biomoléculaires, Substances Naturelles et Réactivité, URAC16, Faculté des Sciences, Semlalia, BP 2390 Bd My Abdellah, 40000 Marrakech, Morocco; b Université Blaise Pascal, Laboratoire des Matériaux Inorganiques, UMR CNRS 6002, 24 Avenue des Landais, 63177 Aubière, France

## Abstract

The title compound, C_16_H_23_Cl_2_NO, was synthesised from β-himachalene (3,5,5,9-tetra­methyl-2,4a,5,6,7,8-hexa­hydro-1*H*-benzocyclo­heptene), which was isolated from the essential oil of the Atlas cedar (*Cedrus Atlantica*). The mol­ecule contains a seven membered ring, which is fused to a five- and a three-membered ring. The five-membered ring has a twisted conformation, whereas the seven-membered ring displays a chair conformation. The dihedral angle between the five- and seven-membered rings is 45.26 (9)°. The absolute structure was established unambiguously from anomalous dispersion effects. In the crystal, mol­ecules are linked into chains propagating along the *b* axis by inter­molecular N—H⋯O hydrogen bonds; an intramolecular N—H⋯O link also occurs.

## Related literature

For the isolation of β-himachalene, see: Joseph & Dev (1968 ▶); Plattier & Teisseire (1974 ▶). For the reactivity of β-himachalene, see: Lassaba *et al.* (1998 ▶); Chekroun *et al.* (2000 ▶); El Jamili *et al.* (2002 ▶); Dakir *et al.* (2004 ▶). For the biological activity of β-himachalene, see: Daoubi *et al.* (2004 ▶). For conformational analysis, see: Cremer & Pople (1975 ▶). 
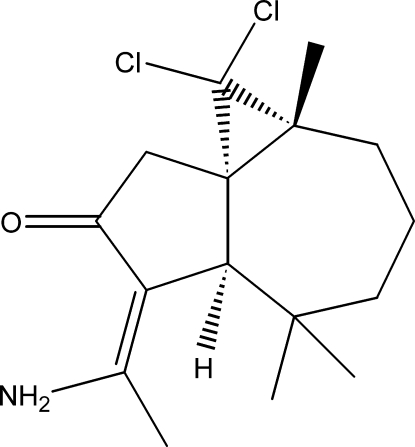


## Experimental

### 

#### Crystal data

C_16_H_23_Cl_2_NO
*M* *_r_* = 316.25
Monoclinic, 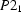

*a* = 7.7570 (7) Å
*b* = 9.7041 (9) Å
*c* = 10.6901 (10) Å
β = 93.432 (3)°
*V* = 803.25 (13) Å^3^
*Z* = 2
Mo *K*α radiation
μ = 0.40 mm^−1^
*T* = 298 K
0.41 × 0.33 × 0.26 mm

#### Data collection

Bruker APEXII CCD diffractometer
4954 measured reflections
2355 independent reflections
2297 reflections with *I* > 2σ(*I*)
*R*_int_ = 0.015

#### Refinement

*R*[*F* ^2^ > 2σ(*F* ^2^)] = 0.028
*wR*(*F* ^2^) = 0.080
*S* = 1.08
2355 reflections
193 parameters
1 restraint
H atoms treated by a mixture of independent and constrained refinement
Δρ_max_ = 0.24 e Å^−3^
Δρ_min_ = −0.22 e Å^−3^
Absolute structure: Flack & Bernardinelli (2000 ▶), 614 Friedel pairs
Flack parameter: −0.02 (5)

### 

Data collection: *APEX2* (Bruker, 2009 ▶); cell refinement: *SAINT-Plus* (Bruker, 2009 ▶); data reduction: *SAINT-Plus*; program(s) used to solve structure: *SHELXS97* (Sheldrick, 2008 ▶); program(s) used to refine structure: *SHELXL97* (Sheldrick, 2008 ▶); molecular graphics: *ORTEP-3 for Windows* (Farrugia, 1997 ▶) and *PLATON* (Spek, 2009 ▶); software used to prepare material for publication: *WinGX* (Farrugia, 1999 ▶).

## Supplementary Material

Crystal structure: contains datablocks I, global. DOI: 10.1107/S1600536811005307/fj2396sup1.cif

Structure factors: contains datablocks I. DOI: 10.1107/S1600536811005307/fj2396Isup2.hkl

Additional supplementary materials:  crystallographic information; 3D view; checkCIF report

## Figures and Tables

**Table 1:** Hydrogen-bond geometry (Å, °)

*D*—H⋯*A*	*D*—H	H⋯*A*	*D*⋯*A*	*D*—H⋯*A*
N1—H2⋯O1^i^	0.85 (3)	2.03 (3)	2.865 (3)	170 (2)
N1—H1⋯O1	0.83 (4)	1.98 (4)	2.672 (3)	140 (3)

Symmetry code: (i) 
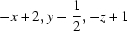
.

**Fig. 1.**
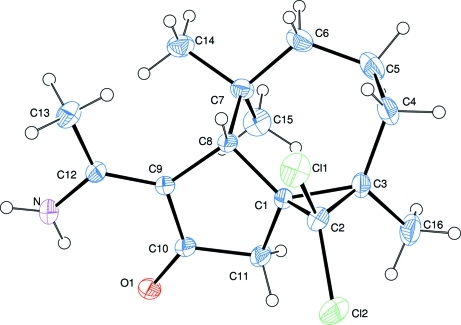
: Molecular structure of the title compound with the atom-labelling scheme. Displacement ellipsoids are drawn at the 30% probability. level. H atoms are represented as small spheres of arbitrary radii.

**Fig. 2.**
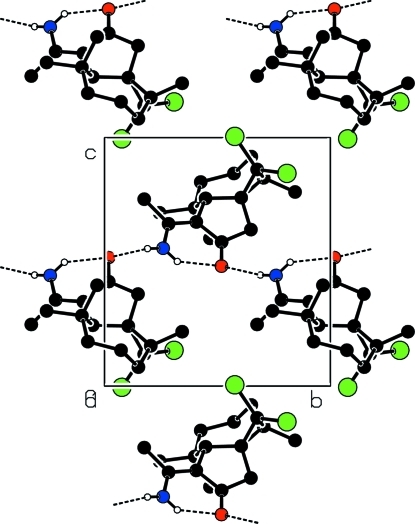
: Partial packing view showing the C—H···O interactions (dashed lines) and the formation of a chain parallel to the c axis. H atoms not involved in hydrogen bonding have been omitted for clarity. [Symmetry code:(i) 2 - y, x-y, z - 1/3]

## supplementary crystallographic information

### Comment

The essential oil of the Alas cedar (Cedrus atlantica) consist mainly (50%) of a bicyclic hydrocarbon called β-himachalene (Joseph & Dev (1968); Plattier & Teisseire(1974)). The reactivity of this sesquiterpene and its derivatives has been studied extensively by our team in order to prepare new products having biological proprieties (Lassaba *et al.*, 1998; Chekroun *et al.*, 2000; El Jamili *et al.*, 2002; Dakir *et al.*, 2004). Indeed, these compounds were tested, using the food poisoning technique, for their potential antifungal activity against phytopathogen Botrytis cinerea (Daoubi *et al.*, 2004). Thus the action of one equivalent of dichlorocarbene, generated *in situ* from chloroform in the presence of sodium hydroxide as base and n-benzyltriethylammonium chloride as catalyst, on β-himachalene produces only (1*S*,3*R*,8*R*)-2,2-dichloro-3,7,7,10- tetramethyltricyclo[6.4.0.0^1,3^] dodec-9-ene (El Jamili *et al.*, 2002). Treatment of the latter by two equivalents of *N*-bromosccinimide (NBS) give (1*S*, 3*R*, 8*R*, 11*R*)-2,2-dichloro-3,7,7,10-tetralethyltricyclo[6.4.0.0^1,3^] dodec-9-en-11-one(Dakir *et al.*, 2004). This enone was treated with the sodium azide in trifluoroacetic acide medium, give with a yield (60%) (1*S*, 3*R*, 8*R*) -9-(1-aminoethylidene)-2,2-dichloro-3,7,7- trimethyltricyclo[6.3.0.0^1,3^]undecan -10-one. The structure of this new product was determined by NMR spectral analysis of 1H, 13 C and mass spectroscopy and confirmed by its single-crystal X-ray structure. The molecule is built up from two fused five-membered and seven-membered rings (Fig. 1). The five-membered ring adopts a twisted conformation,as indicated by Cremer & Pople (1975) puckering parameters Q = 0.2822 (2) Å and φ = 199.2 (4)°. The seven-membered ring displays a chair conformation with QT = 0.7470 (2) Å, θ2 = 27.72 (2)°, φ2 = -51.85 (14)° and φ3 =-78.15 (2)°. In the crystal structure, molecules are linked into chains (Fig. 2) running along the *b* axis by intermolecular N—H···O hydrogen bonds (Table 1) involving the O1 and N atoms. Owing to the presence of Cl atoms, the absolute configuration could be fully confirmed, by refining the Flack parameter (Flack & Bernardinelli (2000)) as C1(S), C3(*R*)and C8(*R*).

### Experimental

To a solution of enone 1 g (3.32 mmol) in 20 ml of trifluoroacetic acid at 10 °C was added with stirring 1 g (15.38 mmol) of NaN3. After being stirred at room temperature for 24 h, the reaction mixture was neutralized with a solution of Na2CO3 (10%) and extracted three time with diethylether (3x20ml). The combined organic phases were dried on Na2SO4, filtred and concentrated at reduced pressure to give the crude product which was chromatographed on a silica gel column with hexane- ether as eluent (20/80) to give 630 mg(1.99 mmol) of (1*S*, 3*R*, 8*R*)-9-(1-aminoethylidene)-2,2-dichloro-3,7,7-trimethyltricyclo [6.3.0.0^1,3^]undecan -10-one.The title compound was recrystallized in diethylether.

### Refinement

except H1 and H2,all H atoms were fixed geometrically and treated as riding with C—H = 0.96 Å (methyl), 0.97 Å (methylene), 0.98Å (methine) with *U*_iso_(H) = 1.2U_eq_ (methylene, methine) or *U*_iso_(H) = 1.5*U*_eq_ (methyl).

### Crystal data

**Table:**

C_16_H_23_Cl_2_NO	*F*(000) = 336
*M**_r_* = 316.25	*D*_x_ = 1.308 Mg m^−^^3^
Monoclinic, *P*2_1_	Mo *K*α radiation, λ = 0.71073 Å
Hall symbol: P 2yb	Cell parameters from 4954 reflections
*a* = 7.7570 (7) Å	θ = 3.4–26.4°
*b* = 9.7041 (9) Å	µ = 0.40 mm^−^^1^
*c* = 10.6901 (10) Å	*T* = 298 K
β = 93.432 (3)°	Prism, colourless
*V* = 803.25 (13) Å^3^	0.41 × 0.33 × 0.26 mm
*Z* = 2	

### Data collection

**Table:**

Bruker APEXII CCD diffractometer	2297 reflections with *I* > 2σ(*I*)
Radiation source: fine-focus sealed tube	*R*_int_ = 0.015
graphite	θ_max_ = 26.4°, θ_min_ = 3.4°
ω and φ scans	*h* = −9→9
4954 measured reflections	*k* = −12→6
2355 independent reflections	*l* = −12→13

### Refinement

**Table:**

Refinement on *F*^2^	Secondary atom site location: difference Fourier map
Least-squares matrix: full	Hydrogen site location: inferred from neighbouring sites
*R*[*F*^2^ > 2σ(*F*^2^)] = 0.028	H atoms treated by a mixture of independent and constrained refinement
*wR*(*F*^2^) = 0.080	*w* = 1/[σ^2^(*F*_o_^2^) + (0.0528*P*)^2^ + 0.0821*P*] where *P* = (*F*_o_^2^ + 2*F*_c_^2^)/3
*S* = 1.08	(Δ/σ)_max_ = 0.001
2355 reflections	Δρ_max_ = 0.24 e Å^−^^3^
193 parameters	Δρ_min_ = −0.22 e Å^−^^3^
1 restraint	Absolute structure: Flack & Bernardinelli (2000), 614 Friedel pairs
Primary atom site location: structure-invariant direct methods	Flack parameter: −0.02 (5)

### Special details

**Table:**

Geometry. All s.u.'s (except the s.u. in the dihedral angle between two l.s. planes) are estimated using the full covariance matrix. The cell s.u.'s are taken into account individually in the estimation of s.u.'s in distances, angles and torsion angles; correlations between s.u.'s in cell parameters are only used when they are defined by crystal symmetry. An approximate (isotropic) treatment of cell s.u.'s is used for estimating s.u.'s involving l.s. planes.

### Fractional atomic coordinates and isotropic or equivalent isotropic displacement parameters (Å^2^)

**Table:**

	*x*	*y*	*z*	*U*_iso_*/*U*_eq_	
H2	1.000 (3)	−0.257 (3)	0.553 (2)	0.042 (6)*	
H1	0.933 (3)	−0.125 (4)	0.502 (3)	0.050 (7)*	
Cl1	0.88492 (8)	0.36063 (7)	0.85606 (7)	0.06081 (18)	
Cl2	0.79425 (7)	0.12962 (6)	1.00496 (4)	0.05055 (16)	
C9	0.7491 (2)	−0.0408 (2)	0.66044 (16)	0.0293 (4)	
O1	0.8629 (2)	0.07077 (17)	0.48287 (13)	0.0448 (4)	
C8	0.6205 (2)	0.0004 (2)	0.75543 (15)	0.0274 (4)	
H8	0.6565	−0.0383	0.8375	0.033*	
C1	0.6443 (2)	0.15793 (19)	0.75843 (15)	0.0297 (4)	
C12	0.8402 (2)	−0.16350 (19)	0.65295 (17)	0.0321 (4)	
C10	0.7797 (2)	0.0713 (2)	0.57971 (17)	0.0333 (4)	
N1	0.9341 (3)	−0.1878 (2)	0.5548 (2)	0.0444 (4)	
C7	0.4310 (2)	−0.0475 (2)	0.71640 (17)	0.0357 (4)	
C3	0.5434 (3)	0.2575 (2)	0.83808 (17)	0.0362 (4)	
C2	0.7312 (2)	0.2285 (2)	0.87178 (18)	0.0363 (4)	
C4	0.4090 (3)	0.2029 (3)	0.9238 (2)	0.0462 (5)	
H4A	0.4656	0.1417	0.9852	0.055*	
H4B	0.3620	0.2797	0.9688	0.055*	
C16	0.4318 (3)	−0.2036 (3)	0.6976 (2)	0.0451 (5)	
H16A	0.5082	−0.2267	0.6333	0.068*	
H16B	0.3171	−0.2344	0.6731	0.068*	
H16C	0.4707	−0.2478	0.7745	0.068*	
C11	0.7012 (3)	0.2010 (2)	0.62976 (17)	0.0386 (5)	
H11A	0.6033	0.2311	0.5759	0.046*	
H11B	0.7855	0.2747	0.6371	0.046*	
C14	0.4943 (4)	0.3966 (3)	0.7812 (2)	0.0543 (6)	
H14A	0.5823	0.4261	0.7276	0.082*	
H14B	0.4834	0.4629	0.8469	0.082*	
H14C	0.3863	0.3887	0.7330	0.082*	
C5	0.2612 (3)	0.1259 (3)	0.8557 (2)	0.0551 (6)	
H5A	0.2260	0.1757	0.7798	0.066*	
H5B	0.1637	0.1242	0.9084	0.066*	
C6	0.3060 (3)	−0.0212 (3)	0.8212 (2)	0.0491 (5)	
H6A	0.1986	−0.0681	0.7975	0.059*	
H6B	0.3545	−0.0657	0.8966	0.059*	
C15	0.3604 (3)	0.0199 (3)	0.5944 (2)	0.0512 (6)	
H15A	0.3540	0.1178	0.6058	0.077*	
H15B	0.2472	−0.0157	0.5721	0.077*	
H15C	0.4358	−0.0003	0.5287	0.077*	
C13	0.8492 (3)	−0.2716 (3)	0.7522 (2)	0.0488 (5)	
H13A	0.9673	−0.2981	0.7700	0.073*	
H13B	0.7832	−0.3504	0.7236	0.073*	
H13C	0.8028	−0.2358	0.8268	0.073*	

### Atomic displacement parameters (Å^2^)

**Table:**

	*U*^11^	*U*^22^	*U*^33^	*U*^12^	*U*^13^	*U*^23^
Cl1	0.0562 (3)	0.0361 (3)	0.0907 (4)	−0.0136 (3)	0.0093 (3)	−0.0118 (3)
Cl2	0.0568 (3)	0.0510 (3)	0.0424 (2)	0.0037 (3)	−0.0094 (2)	−0.0038 (2)
C9	0.0310 (8)	0.0247 (9)	0.0331 (8)	−0.0017 (7)	0.0087 (6)	−0.0004 (7)
O1	0.0579 (9)	0.0357 (8)	0.0439 (7)	−0.0015 (7)	0.0276 (6)	0.0035 (6)
C8	0.0315 (8)	0.0246 (9)	0.0266 (7)	−0.0005 (7)	0.0060 (6)	0.0011 (6)
C1	0.0335 (8)	0.0239 (10)	0.0326 (8)	0.0028 (7)	0.0085 (6)	0.0003 (7)
C12	0.0317 (8)	0.0242 (11)	0.0406 (9)	−0.0021 (7)	0.0043 (7)	−0.0033 (7)
C10	0.0380 (9)	0.0274 (10)	0.0354 (8)	−0.0023 (8)	0.0103 (7)	0.0017 (7)
N1	0.0467 (10)	0.0312 (10)	0.0571 (11)	0.0074 (9)	0.0187 (8)	−0.0038 (10)
C7	0.0328 (9)	0.0380 (11)	0.0365 (9)	−0.0049 (8)	0.0048 (7)	−0.0009 (8)
C3	0.0429 (10)	0.0292 (10)	0.0372 (9)	0.0069 (8)	0.0097 (7)	−0.0042 (8)
C2	0.0410 (10)	0.0262 (10)	0.0419 (9)	−0.0012 (8)	0.0055 (8)	−0.0051 (8)
C4	0.0469 (11)	0.0492 (14)	0.0444 (10)	0.0094 (10)	0.0175 (8)	−0.0066 (10)
C16	0.0449 (11)	0.0412 (13)	0.0493 (11)	−0.0131 (10)	0.0039 (9)	−0.0024 (10)
C11	0.0546 (12)	0.0245 (10)	0.0382 (9)	0.0020 (8)	0.0159 (8)	0.0049 (8)
C14	0.0698 (15)	0.0353 (13)	0.0589 (13)	0.0194 (11)	0.0119 (11)	−0.0001 (11)
C5	0.0365 (10)	0.0652 (17)	0.0656 (12)	0.0064 (12)	0.0196 (9)	−0.0057 (14)
C6	0.0361 (10)	0.0549 (15)	0.0579 (12)	−0.0084 (10)	0.0156 (9)	−0.0035 (11)
C15	0.0459 (12)	0.0577 (16)	0.0484 (11)	0.0006 (11)	−0.0093 (9)	0.0019 (11)
C13	0.0531 (12)	0.0324 (12)	0.0613 (12)	0.0075 (10)	0.0052 (10)	0.0115 (10)

### Geometric parameters (Å, °)

**Table:**

Cl1—C2	1.766 (2)	C4—C5	1.517 (4)
Cl2—C2	1.762 (2)	C4—H4A	0.9700
C9—C12	1.389 (3)	C4—H4B	0.9700
C9—C10	1.418 (3)	C16—H16A	0.9600
C9—C8	1.519 (2)	C16—H16B	0.9600
O1—C10	1.253 (2)	C16—H16C	0.9600
C8—C1	1.539 (3)	C11—H11A	0.9700
C8—C7	1.574 (2)	C11—H11B	0.9700
C8—H8	0.9800	C14—H14A	0.9600
C1—C2	1.515 (3)	C14—H14B	0.9600
C1—C11	1.528 (2)	C14—H14C	0.9600
C1—C3	1.533 (2)	C5—C6	1.519 (4)
C12—N1	1.334 (3)	C5—H5A	0.9700
C12—C13	1.490 (3)	C5—H5B	0.9700
C10—C11	1.509 (3)	C6—H6A	0.9700
N1—H2	0.84 (3)	C6—H6B	0.9700
N1—H1	0.83 (3)	C15—H15A	0.9600
C7—C16	1.528 (3)	C15—H15B	0.9600
C7—C15	1.531 (3)	C15—H15C	0.9600
C7—C6	1.546 (3)	C13—H13A	0.9600
C3—C2	1.506 (3)	C13—H13B	0.9600
C3—C14	1.520 (3)	C13—H13C	0.9600
C3—C4	1.524 (3)		
			
C12—C9—C10	121.23 (16)	C5—C4—H4B	108.7
C12—C9—C8	128.39 (17)	C3—C4—H4B	108.7
C10—C9—C8	110.19 (16)	H4A—C4—H4B	107.6
C9—C8—C1	101.12 (14)	C7—C16—H16A	109.5
C9—C8—C7	112.64 (14)	C7—C16—H16B	109.5
C1—C8—C7	114.08 (16)	H16A—C16—H16B	109.5
C9—C8—H8	109.6	C7—C16—H16C	109.5
C1—C8—H8	109.6	H16A—C16—H16C	109.5
C7—C8—H8	109.6	H16B—C16—H16C	109.5
C2—C1—C11	117.28 (17)	C10—C11—C1	103.64 (15)
C2—C1—C3	59.21 (12)	C10—C11—H11A	111.0
C11—C1—C3	120.82 (16)	C1—C11—H11A	111.0
C2—C1—C8	120.85 (16)	C10—C11—H11B	111.0
C11—C1—C8	107.05 (14)	C1—C11—H11B	111.0
C3—C1—C8	124.93 (16)	H11A—C11—H11B	109.0
N1—C12—C9	120.03 (19)	C3—C14—H14A	109.5
N1—C12—C13	115.56 (19)	C3—C14—H14B	109.5
C9—C12—C13	124.34 (17)	H14A—C14—H14B	109.5
O1—C10—C9	127.82 (19)	C3—C14—H14C	109.5
O1—C10—C11	122.38 (17)	H14A—C14—H14C	109.5
C9—C10—C11	109.76 (15)	H14B—C14—H14C	109.5
C12—N1—H2	120.9 (16)	C4—C5—C6	113.71 (19)
C12—N1—H1	115.2 (19)	C4—C5—H5A	108.8
H2—N1—H1	123 (2)	C6—C5—H5A	108.8
C16—C7—C15	108.37 (18)	C4—C5—H5B	108.8
C16—C7—C6	105.49 (18)	C6—C5—H5B	108.8
C15—C7—C6	109.80 (18)	H5A—C5—H5B	107.7
C16—C7—C8	108.48 (17)	C5—C6—C7	119.6 (2)
C15—C7—C8	112.35 (17)	C5—C6—H6A	107.4
C6—C7—C8	112.04 (16)	C7—C6—H6A	107.4
C2—C3—C14	118.5 (2)	C5—C6—H6B	107.4
C2—C3—C4	118.56 (17)	C7—C6—H6B	107.4
C14—C3—C4	112.69 (18)	H6A—C6—H6B	107.0
C2—C3—C1	59.77 (12)	C7—C15—H15A	109.5
C14—C3—C1	117.47 (17)	C7—C15—H15B	109.5
C4—C3—C1	120.35 (18)	H15A—C15—H15B	109.5
C3—C2—C1	61.01 (12)	C7—C15—H15C	109.5
C3—C2—Cl2	120.86 (14)	H15A—C15—H15C	109.5
C1—C2—Cl2	119.26 (15)	H15B—C15—H15C	109.5
C3—C2—Cl1	119.40 (16)	C12—C13—H13A	109.5
C1—C2—Cl1	121.53 (14)	C12—C13—H13B	109.5
Cl2—C2—Cl1	108.41 (11)	H13A—C13—H13B	109.5
C5—C4—C3	114.04 (18)	C12—C13—H13C	109.5
C5—C4—H4A	108.7	H13A—C13—H13C	109.5
C3—C4—H4A	108.7	H13B—C13—H13C	109.5

### Hydrogen-bond geometry (Å, °)

**Table:**

*D*—H···*A*	*D*—H	H···*A*	*D*···*A*	*D*—H···*A*
N1—H2···O1^i^	0.85 (3)	2.03 (3)	2.865 (3)	170 (2)
N1—H1···O1	0.83 (4)	1.98 (4)	2.672 (3)	140 (3)

Symmetry codes: (i) −*x*+2, *y*−1/2, −*z*+1.
